# Nipah virus infection: A review

**DOI:** 10.1017/S0950268819000086

**Published:** 2019-02-22

**Authors:** Aditi, M. Shariff

**Affiliations:** 1 Department of Microbiology, Guru Teg Bahadur Hospital, Delhi, India; 2 Department of Microbiology, Vallabhbhai Patel Chest Institute, Delhi, India

**Keywords:** Nipah virus, outbreak, review

## Abstract

Nipah virus (NiV) is an emerging bat-borne pathogen. It was first identified 20 years ago in Malaysia and has since caused outbreaks in other parts of South and Southeast Asia. It causes severe neurological and respiratory disease which is highly lethal. It is highly infectious and spreads in the community through infected animals or other infected people. Different strains of the virus show differing clinical and epidemiological features. Rapid diagnosis and implementation of infection control measures are essential to contain outbreaks. A number of serological and molecular diagnostic techniques have been developed for diagnosis and surveillance. Difficulties in diagnosis and management arise when a new area is affected. The high mortality associated with infection and the possibility of spread to new areas has underscored the need for effective management and control. However, no effective treatment or prophylaxis is readily available, though several approaches show promise. Given the common chains of transmission from bats to humans, a One Health approach is necessary for the prevention and control of NiV infection.

## Introduction

Nipah virus (NiV) is an RNA virus belonging to family *Paramyxoviridae*. It belongs to the genus *Henipavirus* which also contains Hendra virus (HeV) and the recently described Cedar virus. Bats are the natural reservoir of Henipaviruses [1]. While Cedar virus has not been found to be pathogenic to any animal, NiV and HeV cause lethal neurologic and/or respiratory disease [2]. NiV is one of the pathogens on the WHO priority list of pathogens likely to cause outbreaks needing urgent research and development action [3]. It first emerged in Malaysia in 1998 and has since caused several outbreaks in South and Southeast Asia. NiV is highly pathogenic to a broad range of mammals and is considered to have pandemic potential due to its zoonotic as well as person to person transmission [4]. The reservoir of infection, *Pteropus* bats, have a worldwide distribution. It is likely that new areas where they reside will be the location of spillover events in the future. A recent outbreak in a new geographical area in Kerala, India is just the latest such event [5]. Research into this disease has been hampered by the relatively small number of cases as well as difficulties in diagnosis. NiV is classified as a Biological safety level 4 (BSL 4) pathogen and access to such laboratories is limited in many countries. Research into epidemiology, modes of transmission and potential prevention and control strategies is needed urgently.

A One Health approach that takes into account humans, domestic and peri-domestic animals and the environment is required to control the disease effectively.

## Methods

We conducted a literature search using the digital archives Pubmed, Google Scholar and the Cochrane library. The following MeSH terms were used: ‘Nipah Virus Infection’, ‘Epidemiology Nipah virus’, ‘Clinical features Nipah virus’, ‘Diagnosis Nipah virus’, ‘Surveillance Nipah virus’, ‘Vaccine Nipah virus’, ‘Nipah virus Malaysia’, ‘Nipah virus Bangladesh’, Nipah virus infection India’ and ‘Nipah virus Philippines’. All literature reviews, original papers and case reports referring to all aspects of NiV origin, modes of transmission, clinical presentation, diagnosis and management were reviewed. The cross-references from these publications were also included. Additionally, epidemiological reports from the WHO and the National centre for disease control (NCDC), India and other public health organisations were assessed for this paper. The aim of this search strategy was to find literature describing the transmission of the disease, diagnosis of infection and control strategies.

## Epidemiology

### Malaysia/Singapore

Human NiV infection was first identified in Malaysia from 1998 to 1999 [6]. The name ‘Nipah’ comes from Sungai Nipah (Nipah River village). A number of cases presenting with fever, headache and reduced consciousness were reported from the state of Perak, Malaysia in September 1998. Initially, four cases tested positive for IgM antibodies against Japanese Encephalitis (JE) and a JE outbreak was declared. Despite the implementation of control measures, the outbreak intensified. By the end of the year, more clusters were reported in Port Dickson District, 300 km south [7]. In March 1999, a new virus (NiV) was isolated from the cerebrospinal fluid (CSF) of a patient from Sungai Nipah village [8]. Eventually, the outbreak caused 283 symptomatic cases and 109 deaths [6]. In March 1999, an outbreak (11 cases, one death) was reported from Singapore among slaughterhouse workers [9].

In these outbreaks, close contact with pigs or pig excreta was shown to be a risk factor [9, 10]. The infected animals themselves showed mild respiratory illness. In Malaysia, large numbers of animals are raised together in pig farms/slaughterhouses, where the outbreak began and animal to animal spread is likely. Culling of over a million pigs followed by disposal by deep burial and decontamination with quick lime, along with other control strategies was successful in controlling the outbreak [11].

Dogs were also found to be commonly infected [12] and dogs dying on farms was found to be another risk factor [10]. There is no evidence of human to human transmission from these outbreaks. Eventually, *Pteropus* bats were shown to be the reservoir of infection in Malaysia [13] which infected the amplifying hosts, pigs, through the consumption of bat-bitten fruit.

### Bangladesh

The epidemiology of NiV is significantly different in Bangladesh. Since 2001, seasonal outbreaks of NiV have occurred in Bangladesh in the winter months, primarily in 20 districts [14] in central and north-western Bangladesh (the ‘Nipah belt’), where the majority of spillover events occur [7]. *Pteropus* bats have been identified as the reservoir [15]. Though contact with pigs has been reported from a majority of patients in Bangladesh, close contact with pigs was found to be a risk factor in one outbreak [16]. Transmission in Bangladesh may occur through various routes. Drinking raw date palm sap is the most common form of transmission of infection from bats to humans [17]. Outbreaks coincide with sap harvesting season (December–May). *Pteropus* bats have been found to visit date palm trees and lick the sap streams being used for collection. Bats may also contaminate the sap collection pots with urine or faeces [18]. Domestic animals may also serve as a route of transmission from bats to humans. Pigs show high seroprevalence against NiV in Bangladesh [19] though they have not been implicated in outbreaks there. This is due to differences in animal husbandry in Bangladesh and Malaysia. Rather than large slaughterhouses, in Bangladesh, individual people own animals in small groups and there is little chance of animal to animal spread. Other animals such as cattle and goats have also been found to be susceptible by seroprevalence studies [19, 20]. Person- to- person spread is an important mode of transmission in Bangladesh and has been identified in all outbreaks. The largest person-to-person outbreak occurred in Faridpur in 2004 [21]. NiV is transmitted via droplet infection [22] and NiV RNA has been detected in the saliva of patients [23]. Other possible pathways include living under a bat roost, where bat urine may infect surroundings. However, no evidence to support this hypothesis has been found [24]. Consumption of bat bitten fruit has also been suspected of being a potential mode of transmission, though definitive evidence has so far been elusive. The primary modes of transmission in Bangladesh have been found to be date palm sap consumption and person-to-person transmission [25].

### India

In India, there was a large outbreak (66 probable cases and 45 deaths) in Siliguri, West Bengal in 2001 and another smaller outbreak (five cases, 100% fatality) in 2007 in Nadia district, West Bengal. These outbreaks were across the border from the Nipah belt in Bangladesh. In May 2018, an outbreak of NiV was declared in Kozhikode and Malappuram districts of Kerala, a southern state in the west coast, which is geographically disconnected from previously affected areas. Date palm sap consumption is not a common practice in this area. There were 18 confirmed cases and 17 deaths as of 1 June 2018 [3]. All cases belonged to the economically productive age group, with no sex differential [26]. In 2001 in Siliguri, the index case remained unidentified but was admitted to Siliguri District Hospital and infected 11 secondary cases, all patients at the hospital. These patients were transferred to other hospitals and further transmission infected 25 staff and eight visitors [27]. The 2007 outbreak consisted of one person who contracted the disease due to consumption of alcohol made from date palm and all the others, including one healthcare worker, acquired the disease from the first case [28]. At least one healthcare professional also contracted the disease in a healthcare setting in the recent outbreak in 2018 [5]. All Indian outbreaks have seen person-to-person transmission. Though the epidemiology of NiV in India is similar to Bangladesh, since only three outbreaks have been reported so far, definitive evidence is unavailable.

### Philippines

An outbreak of NiV infection occurred in the Philippines in 2014. Seventeen cases were confirmed, the case fatality rate was 82%. Ten patients had a history of close contact with horses or of horse meat consumption. Deaths of 10 horses were reported in the same time period, of which nine showed neurological symptoms. However, samples from horses were not tested for NiV. Five patients, including two healthcare personnel, acquired the disease through person to person transmission. This strain was closely related to the Malaysian strain where definite person to person spread had not been previously identified [29]. This suggests the possibility of co-evolution of different strains of NiV in bats or of strain mutation as the likelihood of mutation increases with each spillover event.

## Clinical features

The incubation period of NiV varies from 4 to 21 days. NiV primarily causes acute encephalitis and respiratory illness and is highly fatal. A small percentage of infected people are asymptomatic [10].

A short incubation period is followed by prodromal signs and symptoms such as a fever headache and myalgia [30]. Features of encephalitis develop within a week, with the most common symptoms being altered mental status, areflexia, hypotonia, segmental myoclonus, gaze palsy and limb weakness. Patients deteriorate rapidly and coma and death follow within a few days.

Residual neurological deficits are seen in 20% of survivors and range from fatigue to focal neurological deficits and depression [31]. Some cases of relapsing or late-onset NiV encephalitis have been described [32].

There are some differences in clinical features seen in the Malaysian and Indian outbreaks. A higher mortality rate has been seen in India and Bangladesh (70%) as opposed to Malaysia (40%). Respiratory illness is seen in 70% of patients in India and Bangladesh [27, 33], whereas no significant respiratory involvement was seen in Malaysia [34]. Respiratory involvement may present as a cough, respiratory distress and atypical pneumonia [9,33].

Risk factors for poor prognosis include old age, comorbidities, thrombocytopenia and raised aminotransferases on admission, brainstem involvement and seizures [34].

## Pathogenesis

The Henipaviruses are the only zoonotic paramyxoviruses. They are also exceptional in their broad host range and high case fatality rates. They have a nonsegmented negative-stranded RNA genome consisting of helical nucleocapsids encased in an envelope forming spherical to filamentous, pleomorphic virus particles. Both HeV and NiV have a significantly larger genome than other paramyxoviruses [35]. The genome encodes six structural proteins, the nucleocapsid protein (N), phosphoprotein (P), matrix protein (M), fusion protein (F), glycoprotein (G) and large protein (L) or RNA polymerase, in the order 3′-N-P-M-F-G-L-5′. There are three predicted non-structural proteins, C, V and W which are all encoded by the P gene [36].

The virus enters its host through the oro-nasal route and causes infection. Since human tissues have only been studied from the terminal stages of the disease, the site of initial replication is unknown. However, high concentrations of antigen found in lymphoid and respiratory tissues indicate these tissues as probable sites of initial replication [37]. Early viraemia leads to the spread of the virus and is followed by secondary replication in the endothelium. The glycoprotein G of NiV binds to the cellular receptor Ephrin-B2 (alternate receptor Ephrin-B3) which is expressed on endothelium and smooth muscle cells in high levels in the brain, followed by lungs, placenta and prostate, along with blood vessels in various other tissues [38]. This receptor distribution explains the clinical and pathological features seen in this disease. Ephrin-B2 plays a critical role in the migration of neuron precursors during embryogenesis [39]. Therefore, it is highly conserved between different classes of animals and receptor similarity with bats and pigs approaches 95–96% [40]. This leads to the wide host range seen with NiV. The central nervous system is invaded primarily by the haematogenous route, though evidence of direct invasion through olfactory nerves has been seen in a porcine model [41]. The high lethality of NiV is attributed to its evasion of the innate immune response. The P gene products have been shown to inhibit interferon activity [42]. Another study has demonstrated inhibition of interferon production, with little effect on interferon signaling due to NiV infection [43].

In Malaysia, two major strains of NiV were found circulating in pigs, one in the northern part of the country and the other in the south and at least two introductions of the virus into pigs are thought to have occurred [44]. The NiV strain circulating in Bangladesh and India is different from the Malaysian strains and has a genome six nucleotides longer [23, 28]. These different strains are likely to have coevolved separately with their reservoir hosts, the bats [45]. This may explain the differences observed in clinical and epidemiological features.

## Diagnosis

Specimens for virus detection may be collected from symptomatic patients or at post-mortem examination. Specimens for serological testing should be collected late in the course of infection, 10–14 days after onset. The NCDC, India, recommends throat swabs (in viral transport medium), urine, blood and/or CSF for diagnosis. Samples must be collected safely and transported in triple container packing at 2–8 °C. Storage at −20 °C is recommended beyond 48 h of collection. Processing of the clinical samples requires a BSL 4 facility. However, virus inactivation by sample irradiation may be an effective technique to make the samples safe to use in a BSL-2 laboratory [27, 46].

### Direct detection of the agent

The best test for direct detection is polymerase chain reaction (PCR) due to its high sensitivity, specificity and the rapidity with which results can be reported. Specimens that may be used include tissue samples, swabs, CSF and urine. Diagnosis by direct detection in animals may be difficult, as virus detection has low sensitivity.

#### PCR

Conventional PCR targeting the N (nucleocapsid protein) gene has been developed by the US Centers for Disease Control and Prevention (CDC) [20]. NiV RNA can be identified by Real-Time PCR (RT-PCR) from respiratory secretions, urine or cerebrospinal fluid. These tests are highly sensitive and specific and are used commonly for diagnosis. A TaqMan probe-based assay developed in 2004 [47] detects the N gene and has a very high sensitivity of ~1 pfu. It is specific for NiV RNA and can be used for diagnosis during an outbreak. A SYBR Green-based assay targeting a different region of the N gene [48] has also been developed. It has lower sensitivity (~100 pfu) and detects HeV as well.

#### Immunohistochemistry

Formalin-fixed tissue may be used for immunohistochemistry. Since the site of viral replication is vascular endothelium, a wide range of tissues may be used including brain, lung, spleen, kidney and lymph nodes. Uterus, placenta and products of conception are also analysed in pregnant animals. Previously, convalescent human serum was used for immunohistochemistry. This has now been replaced by rabbit serum against NiV [49].

#### Virus Isolation

Virus isolation from respiratory secretions, urine, cerebrospinal fluid or other tissue specimens must be done in a BSL-4 laboratory. The cell line of choice for both NiV and HeV is the Vero cell line. Pteroid bat cell lines have also been developed [50]. Cytopathic effects can be observed within 3 days. The cells form syncytia and subsequently, there is the formation of punctate holes in the monolayer as the syncytia lift from the surface [49]. NiV forms larger syncytia than HeV and the differences in nucleus distribution in the syncytia can be used to differentiate the two [51]. Definitive identification of the virus from cell culture can be done by PCR or immunohistochemistry.

Other tests that may be used include sequencing, which is used for virus characterisation and electron microscopy. However, they are infrequently available and are inappropriate for primary diagnosis.

### Antibody detection

IgM antibody in serum or CSF is used for diagnosis. Detection of IgG antibodies is a good test for surveillance in humans and for the identification in reservoir animals during epidemiological investigations. It has also been used for diagnosis in humans during outbreaks.

#### ELISA

It is the most commonly used test for serological diagnosis due to its high sensitivity, rapidity, ease and safety of use. ELISAs for the detection of IgG and IgM developed by the CDC were used in the confirmation of diagnosis in Malaysia [49]. It has since been used for surveillance in Bangladesh during NiV outbreaks [22]. Other tests based on recombinant proteins have been developed using the more conserved N antigen [52, 53]. IgM antibodies have been found to be detectable in 50% patients on day 1 of illness, while 100% of patients show IgG positivity after day 18. IgG positivity persists for several months [54].

#### Serum Neutralisation Test

This is considered the gold standard test but requires the use of a BSL-4 laboratory. In this test, test sera are incubated with the virus and then allowed to infect Vero cells. Positive sera block the development of cytopathic effects and tests can be read at 3 days. A modified neutralisation test which can be read at 24 h has been developed [55]. Here, the virus-serum mixture is removed after a period of adsorption and immunostaining is used for virus detection.

Pseudo typed viruses can be used to perform a surrogate neutralisation test. A pseudo typed virus is an enveloped virus with one or more foreign envelope proteins. These viruses can be handled safely in the BSL-2 laboratory but contain NiV envelope proteins capable of being neutralised by positive sera [56].

In non-endemic areas where NiV outbreaks have not occurred, a high index of suspicion is required for rapid identification and containment of an outbreak. The NCDC, India has issued guidelines on the definitions of a Suspected, Probable and Confirmed case of NiV infection (Table 1) which have been used effectively to control known outbreaks in India.

**Table 1.** Surveillance case definitions for Nipah virus infection as defined by the National Centre for Disease Control, India

Case type	Description
Suspect case	Person from a community affected by a Nipah outbreak who: Fever with new onset altered mental status or seizure and/or Fever with headache and/or Fever with cough or shortness of breath
Probable case	Suspect case-patients/s who resided in the same village where confirmed case-patient/s were living during the outbreak period and who died before diagnostic specimens could be collected. OR Suspect case-patients who came in direct contact with confirmed case-patients in a hospital setting during the outbreak period and who died before complete diagnostic specimens could be collected.
Confirmed case	Suspected case who has laboratory confirmation of Nipah virus infection either by: Nipah virus RNA identified by PCR from respiratory secretions, urine or cerebrospinal fluid. Isolation of Nipah virus from respiratory secretions, urine or cerebrospinal fluid.

## Management and control

Patients must be isolated and rigorous infection control practices implemented. Treatment of NiV infection is primarily supportive- maintenance of airway, breathing and circulation. Fluid and electrolyte balance is maintained. Patients with severe pneumonia and acute respiratory failure must be supported by mechanical ventilation. Invasive mechanical ventilation is preferred.

### Antiviral Chemotherapy

Ribavirin, which is effective against other Paramyxoviruses (such as Respiratory Syncytial Virus) was used to treat infected patients in Malaysia. Chong *et al*. [57] reported a decrease in mortality while Goh *et al*. [34] found no decrease during the same outbreak. Ribavirin has since been tested in animal models and found to be ineffective [58]. However, in the absence of effective antivirals, the NCDC recommends the use of oral or parenteral Ribavirin for all confirmed cases. Ribavirin is not recommended for chemoprophylaxis [26]. Acyclovir was used in Singapore but whether it was effective is unclear [9]. Chloroquine was reported to be effective in cell culture but failed to prevent death in a hamster model in isolation or in combination with Ribavirin [59]. The natural ligands of Ephrin-B2, as well as soluble Ephrin-B2, have been shown to be effective *in vitro* [60]. Favipiravir, a drug licensed in Japan for treatment of Influenza, has been shown to be effective in a hamster model [61]. Neutralizing human monoclonal antibody has been found to be effective in a non-human primate model [62]. Use of anti-G and anti-F monoclonal antibodies in an emergency setting is approved in India. Patients are discharged only after a negative RT-PCR result on throat swab/blood is obtained. Period of communicability is unknown but presumed to be 21 days. Therefore, discharged patients are also advised to remain in isolation until 21 days after confirmation of infection [26].

### Surveillance

Disease surveillance is carried out regularly in the Nipah belt in Bangladesh. Surveillance activities consist of event-based and sentinel surveillance. Print and electronic media surveillance is carried out in 10 national newspapers and eight national news channels and hotlines have been set up for healthcare personnel to report outbreaks. Suspected outbreaks and deaths due to unknown causes are rapidly identified through these methods. Under sentinel surveillance clusters of encephalitis are investigated. Clusters are defined as two or more cases within 21 days and half an hour's walk from each other [16]. A team of epidemiologists from the Institute of Epidemiology, Disease Control and Research, Bangladesh investigates any identified clusters. The team identifies suspected human cases, potential animal sources of infection, behavioural factors contributing to infection and environmental contamination. Surveillance is an important part of disease management and should be instituted in areas that have seen outbreaks like India and other countries in the region.

### Prevention

Efforts towards prevention have primarily focussed on the prevention of contamination of date palm sap, increasing awareness about the dangers of consuming date palm sap and prevention of person-to-person spread. The use of skirts to cover the sap producing areas of date palm trees has been found to effectively prevent contact with bats [63]. In 2015, a study assessing the behaviour of people consuming raw date palm sap, found that awareness of NiV was very low among them and even people who were aware of it were just as likely to consume it as people who did not [64]. A randomised controlled trial assessing behaviour change communication intervention conducted in 2017 found that disseminating a message encouraging consumption of safe sap reduced exposure to potentially contaminated sap while a message discouraging consumption of sap at all did not [65]. The WHO advisory during an ongoing outbreak includes avoiding exposure to pigs and bats and consumption of bat-bitten fruits or raw date palm sap/toddy/juice. In order to reduce the risk of animal- to- human transmission gloves and other protective clothing should be worn while handling sick animals or their tissues and during slaughtering and culling procedures.

Prevention of person-to-person transmission includes the implementation of infection control practices such as isolation of patients, use of personal protective equipment and good hand hygiene practices. Contacts identified through contact tracing are tested and kept under observation until they test negative. Hospital surfaces have been found to be contaminated by NiV around patients [66]. Healthcare facilities must institute and ensure compliance to standard infection prevention and control measures when caring for suspected or confirmed cases of NiV infection. Health care workers exposed to a suspected NiV patient should inform the authorities and undergo testing for NiV [3]. Contacts of infected patients are counseled to avoid prolonged close personal contact with patients. Funeral practices requiring direct contact with the remains are discouraged.

### Vaccines

A number of vaccine strategies have been developed for NiV, several of which have been tested in animal models. The most studied approach has been a subunit vaccine based on G glycoprotein (sG) of NiV and HeV. HeV-sG elicits a cross-protective immune response against both HeV and NiV [67]. It has now been developed into a horse vaccine against HeV called Equivac which is registered in Australia. Virus vector-based recombinant vaccines have also been developed. These recombinant viruses express the F or G glycoproteins on their surface [68, 69]. A mammalian cell-derived virus-like particle vaccine has also been produced [70]. All these approaches have produced complete protection against oro-nasal NiV challenge after a single dose in various animal models. The success of the sG vaccine in horses and of the VSV vectored Ebola vaccine (rVSV-ZEBOV) make these two approaches attractive for eventual use in humans.

## Conclusion

NiV has emerged as a deadly zoonotic disease. Bats, the natural reservoir of the virus, are effective at virus dissemination and human outbreaks continue to be reported regularly. Due to the worldwide distribution of bats, outbreaks in new areas are likely to occur. The high case fatality rate and acute course of disease make the infection difficult to diagnose. This is further compounded by the lack of easily available low-cost diagnostic tests and facilities equipped to handle viral samples. Effective treatment and prophylaxis are unavailable due to a lack of studies in human subjects because the overall case burden is small and the course of infection is acute. The recent outbreak in India highlights the possibility of potential spillover events in areas where currently known risk factors do not exist. Establishment of surveillance systems for NiV is necessary, particularly in South and Southeast Asia. There is an urgent need for countries in South and Southeast Asia to work together to strengthen surveillance systems in order to monitor spillover events and prevent transmission. A better understanding of bat ecology and the causes of spill-over events, the development of effective treatment and prophylaxis for humans and animals and strengthening of surveillance systems to prevent outbreaks is required to curb the threat posed by NiV.
